# 

**Published:** 1926

**Authors:** 


### The Bristol Medico-Chirurgical Journal

A JOURNAL OF THE MEDICAL SCIENCES FOR THE WEST OF ENGLAND AND SOUTH WALES.

PUBLISHED UNDER THE AUSPICES OF

*THE BRISTOL MEDICO-CHIRURGICAL SOCIETY.*

EDITOR:

PATRICK WATSON-WILLIAMS, M.D.

WITH WHOM ARE ASSOCIATED

J. LACY FIRTH, M.S., M.D., CAREY COOMBS, M.D.,
E. W. HEY GROVES, M.S., F.R.C.S., B.Sc.
E. WATSON-WILLIAMS, M.C., Ch.M., F.R.C.S.E.

J. A. NIXON, C.M.G., M.D., Assistant Editor.

EDITORIAL SECRETARY:

A. L. FLEMMING, M.B., B.Ch.

---

"Scire est nescire, nisi id me

Scire alius scierit."

---

*VOL. XLIII.*

BRISTOL: J. W. ARROWSMITH LTD.
London: J. W. Arrowsmith (London) Ltd.

1926.

ARMY MEDICAL LIBRARY

296935

WASHINGTON, D.C.

WI
BR323

### Contents of Vol. XLIII.

*Page*

The Ætiology of Cardiac Disease. By CAREY F. COOMBS, M.D., F.R.C.P. Lond. .. .. .. .. .. .. .. .. 1

Suprarenal Virilism, with an Account of a Case. By EDWARD FAWCETT, M.D., C.M., F.R.S., GEOFFREY HADFIELD, M.D., M.R.C.P. Lond., and PERCY PHILLIPS, M.Sc., M.B., B.Ch. Bristol .. .. .. .. 20

The Ocular Signs of Migraine. By E. R. CHAMBERS, F.R.C.S. .. .. 29

Uveo-Parotitic Paralysis. A Report of a Case and some Account of Previous Instances. By GEORGE PARKER, M.A., M.D. Cantab. .. 73

Polyneuritis preceded by Inflammation of the Parotid, Lachrymal and Mammary Glands. By BEATRICE ROGERS, L.R.C.P., L.R.C.S. Edin. and J. HERVEY BODMAN, M.D. Lond. .. .. .. .. .. 84

The Operative Treatment of Meningitis. By E. WATSON-WILLIAMS, M.C., Ch.M., F.R.C.S.E. .. .. .. .. .. .. .. .. 91

Rigid Rest in Rheumatic Carditis. By J. A. NIXON, C.M.G., M.D. Cantab., F.R.C.P. .. .. .. .. .. .. .. .. .. 96

Personality—A Review. By Professor C. LLOYD MORGAN, F.R.S. .. 104

The Relation of the Autonomic Nervous System to Clinical Medicine. By J. R. CHARLES, M.A., M.D. Cantab., F.R.C.P. .. .. .. .. 127

The Pathology of Cervical Erosion. By GEOFFREY HADFIELD, M.D., M.R.C.P. Lond. .. .. .. .. .. .. .. .. 151

The Investigation of Surgical Cases of Renal Disease. By D. G. C. TASKER, B.Sc., M.S., F.R.C.S. .. .. .. .. .. .. .. 161

A Case of Severe Herpes Zoster and Varicella occurring simultaneously in an Aged Patient. By R. E. OVERTON, M.R.C.S., L.R.C.P. .. .. 169

Kala azar. Notes on the Disease and its Treatment. By ERSKINE FARAKER, M.R.C.S., L.R.C.P. .. .. .. .. .. .. 183

The Treatment of Lachrymal Obstruction. By A. E. ILES, O.B.E., M.B., F.R.C.S. Eng. .. .. .. .. .. .. .. .. .. 191

The Use of Insulin in Surgery. By J. A. NIXON, C.M.G., M.D. Cantab., F.R.C.P. .. .. .. .. .. .. .. .. .. 199

The Treatment of Repressed Memories by Hypnotism. By PERCIVAL S. CONNELLAN, M.R.C.S. .. .. .. .. .. .. .. 209

REVIEWS OF BOOKS .. .. .. .. .. .. 38, 108, 174, 217

EDITORIAL NOTES .. .. .. .. .. .. .. 47, 119, 178, 227

MEETINGS OF SOCIETIES .. .. .. .. .. .. 53, 122, 231

OBITUARY .. .. .. .. .. .. .. .. .. 59, 235

THE MEDICAL LIBRARY OF THE UNIVERSITY OF BRISTOL 62, 124, 180, 240

LOCAL MEDICAL NOTES .. .. .. .. .. .. 64, 126, 182, 242

He leaves a widow, two sons, the elder being the Rev. Canon H. A. Prichard, of New York, the younger Lieut. N. Prichard in the Royal Navy, and three daughters, and fifteen grandchildren to mourn his loss. Few men have been more universally beloved than was Arthur Prichard.

### The Library of the Bristol Medico-Chirurgical Society.

*The following publications have been received since the publication of the List in December,* 1925.

*February,* 1926.

| | | |
|---|---|---|
| Dr. D. S. Davies (1) .. .. .. .. | 1 | volume. |
| J. Wright & Sons Ltd. (2) .. .. .. | 1 | ,, |
| Dr. P. Watson-Williams (3) .. .. .. | 1 | ,, |
| Surgeon-General of the United States Army (4) | 1 | ,, |
| Joint War Committee of the British Red Cross Society (5) .. .. .. .. .. | 1 | ,, |
| Michell Clarke Memorial Fund (6) .. .. | 5 | volumes. |
| Munro Smith Memorial Fund (7) .. .. | 4 | ,, |

Unbound periodicals have been received from Professor Hey Groves and Dr. Carey Coombs.

### THE ONE HUNDRED AND FIFTEENTH LIST OF BOOKS.

The figures in round brackets refer to the figures after the names of the donors. The books to which no such figures are attached have either been bought from the Library Fund or received through the *Journal*.

**Brockbank, E. M.** .. *Incapacity or Disablement in its Medical Aspects* 1926

**Cameron, S. J., and Hewitt, J.** *Uterine Hæmorrhage* .. .. .. .. .. .. 1926

**Crookshank, F. G.** .. *Migraine and other Common Neuroses* .. .. .. 1926

| | | | |
|---|---|---|---|
| **Drew, H. R.** | *Malignant Diseases of the Testicle* | (6) | 1925 |
| **Easterbrook, C. C.** | *Mental Invalids* | | 1926 |
| **Ewing, J.** | *Neoplastic Diseases* | 2nd Ed. (7) | 1922 |
| **Geddes, G.** | *Puerperal Septicœmia* | | 1923 |
| **Goodhart, Sir J. F., and Still, G. F.** | *Diseases of Children* | 12th Ed. (7) | 1925 |
| **Hornibrook, F. A.** | *Physical Fitness in Middle Life* | | 1925 |
| **Joslin, E. P.** | *Treatment of Diabetes Melitus* | 3rd Ed. | 1924 |
| **Lewis, T.** | *Mechanism and Graphic Registration of the Heart Beat* | 3rd Ed. (7) | 1925 |
| **McCrae, T** | *Osler's Principles and Practice of Medicine* | 10th Ed. (6) | 1925 |
| **MacFarland, J.** | *Surgical Pathology* | (6) | 1924 |
| | *Medical Department of the United States Army in the World War.* Vol. XV. | (4) | 1925 |
| **Morison, R., and Saint, C. F. M.** | *An Introduction to Surgery* | | 1925 |
| **Pauchet, V.** | *Practical Surgery.* Vols. I. and II. | (7) | 1924 |
| **A Pharmacist** | *Principal Drugs* | | 1926 |
| **Poulton, E. P.** | *Taylor's Practice of Medicine* | 13th Ed. (7) | 1925 |
| **Ryall, E. C.** | *Operative Cystoscopy* | (6) | 1925 |
| **Siemens, H. W., and Barker, L. F.** | *Race Hygiene and Heredity* | (1) | 1924 |
| **Steindler, A.** | *Operative Orthopedics* | | 1925 |
| **Thomson, H. C., and Riddoch, G.** | *Diseases of the Nervous System* | 4th Ed. | 1926 |
| **Watson-Williams, P.** | *The Semon Lecture,* 1925 | (3) | 1925 |
| **Wright's of Bristol** | *Centenary Souvenir* | (2) | 1925 |

### TRANSACTIONS, REPORTS, JOURNALS, ETC.

| | | |
|---|---|---|
| Annual Report of the Chief Medical Officer of the Board of Education | | 1925 |
| British Journal of Surgery, January | | 1926 |
| Report of the Medical Research Council, 1924-25 | | 1925 |
| Reports of the Joint War Committee of the British Red Cross Society, 1914-19 | (5) | 1921 |

### Local Medical Notes.

EXAMINATION RESULTS.

**University of Bristol.**—Students of the University have recently passed the following examinations :—

M.B., Ch.B (*Part I.*).—*Final Examination:* C. Batt, W. P. Brinckman, A. M. Critchley, T. F. Hewer (with distinction in Materia Medica, Pharmacy, Pharmacology and Therapeutics), R. Jackson, J. E. Martin, J. R. E. Sansom, M. Srisvasti, D. M. H. Tripp (with distinction in Materia Medica, Pharmacy, Pharmacology and Therapeutics), E. R. Wide. *Part II.* (*Completing Examination*) : (*Honours*) C. B. Perry (with distinction in Obstetrics). *Pass :* G. W. R. Bishop, R. V. Cooke, F. S. Dymond, F. R. Gedye (with distinction in Pathology, Forensic Medicine and Toxicology), A. P. Gorham, T. P. Lalonde, G. M. Minifie, L. B. Phillips, A. S. Prowse. *In Group II. only :* G. L. Feneley, C. France-Hayhurst.

Ch.M.—*In Gynæcology :* H. L. Shepherd.

B.D.S.—*3rd Examination :* E. C. Browne.

Diploma in Dental Surgery.—*Final Examination :* R. Lonnon. *2nd Professional Examination :* P. J. Whiteman, A. E. Wilkins. *Pass* (*in Dental Metallurgy and Dental Mechanics*) (*Completing Examination*) *:* J. H. Edwards. *In Dental Mechanics* (*Completing Examination*) : D. M. Cox. *In Dental Materia Medica* (*Completing Examination*) *:* P. H. Slater. *In Dental Mechanics and Dental Metallurgy only :* E. J. Tanner, G. W. Vowles. *In Dental Metallurgy only :* A. L. Gilchrist.

Conjoint Board.—M.R.C.S., L.R.C.P. (*Completing Examination*) : R. V. Cooke, A. P. Gorham, T. P. Lalonde.

APPOINTMENTS.

Dr. J. A. Nixon has been appointed Honorary Consulting Physician and Dr. R. S. S. Statham Honorary Obstetric Physician to the Clifton Dispensary.

### Bristol Medico-Chirurgical Society.

---

### President.
T. CARWARDINE, M.S. Lond.

### President-Elect.

A. L. FLEMMING, M.B., B.Ch.

### Committee.

*Ex-Officio*
- J. ODERY SYMES. M.D. Lond. (Retiring President).
- P. WATSON-WILLIAMS, M.D. Lond. (Editor of *Journal*).
- C. K. RUDGE (Hon. Librarian).
- A. L. FLEMMING, M.B., Ch.B. Brist. (Editorial Secretary).

*Elected.*

F. LACE } Ex-Presidents.
J. A. NIXON, C.M.G., B.A., M.D. Cantab. } Ex-Presidents.
D. C. RAYNER.
C. FERRIER WALTERS.
E. W. HEY GROVES, M.D., M.S. Lond.
A. J. WRIGHT, M.B., B.S. Lond.
H. L. ORMEROD, M.D. (R.U.I.).
W. VINCENT WOOD, M.C., B.A. Cantab.

### Honorary Secretary.
S. V. STOCK, M.B., B.S. Lond., M.B., B.Ch. Brist.

Medical Practitioners wishing to join the Society are requested to communicate with the Hon. Secretary, Mr. S. V. STOCK, 1 Mortimer Road, Clifton. The Annual Subscription is £1 11s. 6d.

---

*Extracts from Journal By-laws.*

4.—The *Journal* shall be delivered free to Subscribers and also to Members of the Society whose subscriptions are not in arrear.

6.—The Annual Subscription to the *Journal* is 10/6.

---

Communications intended for insertion in the *Journal* should be sent to the Editor, Dr. P. WATSON-WILLIAMS, 2 Rodney Place, Clifton, Bristol.

Books for Review and Exchange Journals should be sent to the Assistant-Editor, *Bristol Medico-Chirurgical Journal*, Medical Library, The University, Bristol.

Communications referring to the delivery of the *Journal* should be sent to the Editorial Secretary, A. L. Flemming, 48 Pembroke Road, Clifton, Bristol, to whom Subscriptions are to be paid *in advance*.

Cases for Binding Vols. I—XLII are now ready, and may be obtained, price 1/9 each (postage extra), upon application to J. W. ARROWSMITH LTD., Quay Street, Bristol; or the *Journal* will be bound, including Case, for 7/- per volume.

### Bristol Medico-Chirurgical Society.

### PAST PRESIDENTS.

1874—75. FREDERICK BRITTAN, M.D., B.A. Dub.
1875—76. ROBERT WILLIAM COE.
1876—77. SAMUEL MARTYN, M.D. Ed.
1877—78. AUGUSTIN PRICHARD, M.D. Berlin.
1878—79. HENRY EDWARD FRIPP, M.D. Würzburg.
1879—80. WILLIAM MICHELL CLARKE.
1880—81. JOSEPH GRIFFITHS SWAYNE, M.D. Lond.
1881—82. EDWARD LONG FOX, M.D. Oxon.
1882—83. JAMES GEORGE DAVEY, M.D. St. And.
1883—84. WILLIAM JOHNSTONE FYFFE, M.D., B.A. Dub.
1884—85. GEORGE FORSTER BURDER, M.D. Aberd.
1885—86. WILLIAM HENRY SPENCER, M.D., M.A. Cantab.
1886—87. FRANCIS POOLE LANSDOWN.
1887—88. LEMUEL MATTHEWS GRIFFITHS.
1888—89. ROBERT SHINGLETON SMITH, M.D., B.Sc. Lond.
1889—90. NELSON CONGREVE DOBSON, Ch.M. Bristol.
1890—91. SAMUEL HENRY SWAYNE.
1891—92. FRANCIS RICHARDSON CROSS, M.B.Lond., LL D.Bristol.
1892—93. EDWARD MARKHAM SKERRITT, M.D., B.S., B.A. Lond.
1893—94. JAMES GREIG SMITH, M.B., C.M., M.A. Aberd., F.R.S.E.
1894—95. ALFRED JAMES HARRISON, M.B. Lond.
1895—96. ARTHUR WILLIAM PRICHARD.
1896—97. ALFRED EDWARD AUST LAWRENCE, M.D., C.M. Aberd.
1897—98. JOHN EDWARD SHAW, M.B., C.M. Ed.
1898—99. ROBERT ROXBURGH, M.B. Ed.
1899—00. WILLIAM HENRY HARSANT.
1900—01. DAVID SAMUEL DAVIES, M.D.,Lond.
1901—02. BARCLAY JOSIAH BARON, M.B., C.M. Ed.
1902—03. GEORGE MUNRO SMITH, M.D. Bristol.
1903—04. JAMES PAUL BUSH, C.M.G., C.B.E., Ch.M. Bristol.
1904—05. REGINALD EAGER, M.D. Lond.
1905—06. JOHN DACRE.
1906—07. JAMES TAYLOR.
1907—08. HENRY WALDO, M.D., C.M. Aberd.
1908—09. JOHN MICHELL CLARKE, M.D., M.A. Cantab.
1909—10. JAMES SWAIN, C.B., C.B.E., M.D., M.S. Lond.
1910—11. BERTRAM MITFORD HERON ROGERS, M.D., B.Ch., B.A. Oxon.
1911—12. CHARLES ALEXANDER MORTON.
1912—13. WALTER CARLESS SWAYNE, M.D., B.S. Lond. and Bristol.
1913—14. PATRICK WATSON-WILLIAMS, M.D. Lond.
1914—15. WILLIAM HARRY CHRISTOPHER NEWNHAM, M.A., M B. Cantab.
1915—16 GEORGE PARKER, M.A., M.D. Cantab.
1916—17 FRANCIS HENRY EDGEWORTH, M.A., M.D. Cantab., D.Sc. Lond.
1917—18 FREDERICK LACE.
1918—19 ROBERT GUTHRIE POOLE LANSDOWN, M.D., B.S. Durh.
1919—20 I. WALKER HALL, M.D., Ch.B. Vict.
1920—21 LEOPOLD ERNEST VALENTINE EVERY-CLAYTON, M.D. Lond.
1921—22 CYRIL HUTCHINSON WALKER, M.B., B.A. Cantab.
1922—23 JOHN ALEXANDER NIXON, C.M.G., B.A., M.D. Cantab.
1923—24 JOHN LACY FIRTH, M.D., M.S. Lond.
1924—25 J. ODERY SYMES, M.D. Lond.

1926.

---

### LIST OF SUBSCRIBERS

TO THE

### Bristol Medico-Chirurgical Journal.

---

### HONORARY MEMBERS OF THE SOCIETY.

OWEN, SIR ISAMBARD, D.C.L., M.D. Abbey Gate, Beach Road, Weston-super-Mare.

RUDGE, C. KING ... ... ... ... ... ... 145 Whiteladies Rd., Clifton.

SWAIN, JAMES, *C.B.*, *C.B.E.*, M.D., M.S. Wyndham House, Easton-in-Gordano.

---

### SUBSCRIBERS.

*Those marked * are Members of the Society.*

*ACKLAND, W. R. ... ... ... ... ... ... ... ... 5 Rodney Place, Clifton.
*ADAMS, A. W., M.B., B.Sc. Lond. ... ... ... 9 Mortimer Road, Clifton.
ADYE, W. J. H. ... ... ... ... ... ... ... ... St. Margaret's, Bradford-on-Avon.
ALFORD, H. J., M.D. ... ... .. ... ... ... 4 Park Terrace, Taunton.
*ALEXANDER, A., M.A. ... ... ... ... ... ... .. 23 Manilla Road, Clifton.
*ALEXANDER, D. A., M.B., Ch.B. Vict. ... ... 30 Berkeley Square, Clifton.
*ASKINS, R. A., M.D., B.Ch., B.A.O. Dubl.... Guildhall, Bristol.
*AUBREY, T., M.B. Lond. ... ... ... ... ... Bitton, near Bristol.

*BAKER, LILY A., M.D., B.Ch., B.A.O., R.U.I.... 21 Victoria Square, Clifton.
*BARBER, M. C., M.B., Ch.B. Brist. ... ... ... Ingledene, High Street, Staple Hill Bristol.
*BARKER, G. H., M.D., B.Sc. Lond. ... ... ... 124 Redland Road, Bristol.
*BARTHOLOMEW, E. U. ... ... ... . ... ... 12 Lansdown Place, Clifton.
*BENSON, ELIZABETH, M.B., Ch.B. Brist. ... 4 Caines Road, Horfield.
*BERGIN, F. G. ... ... ... ... ... ... ... ... 31 Oakfield Road, Clifton.
BERNARD, C. ... ... ... ... ... ... ... .. ... 564 Fishponds Road, Fishponds, Bristol.
BERRY, F. C., M.D., B.Ch., B.A. Dub. ... ... Locarno, Burnham, Somerset.
*BIRRELL, J. A., M.B., B.S. Lond. ... ... 51 Sussex Place, Ashley Road.
BLACKETT, J. F., M.D., B.S. Lond. ... ... ... Hamsterley, Bloomfield Park, Bath.
*BLAGG, ARTHUR F., M.D. Durh.... ... ... ... 6 Upper Belgrave Road, Clifton.
*BLATHWAYT, A. de V. ... ... ... ... ... ... 6 Brock Street, Bath.
BLETCHLY, Dr. G. Playne, M.B.... .. ... ... Hazelwood, Nailsworth, Glos.

### LIST OF SUBSCRIBERS.

| | |
|---|---|
| *Bodman, C. O., M.D. Durh. | 17 Upper Belgrave Road, Clifton. |
| *Bodman, F. H., M.B., Ch.B. Brist. | 170 Cheltenham Road. |
| *Bodman, J. Hervey, M.D. Lond. | 9 Whiteladies Road, Clifton. |
| *Bowker, G. E., M.D. Edin. | 11 North Parade, Bath. |
| *Bradbeer, E. G. | 32 Linden Road, Westbury Park. |
| *Bray, G., Lt.-Col., *D.S.O.* | 26 Beaconsfield Road. |
| *Brentnall, T. C. | Mendip Hills Sanatorium, Wells. |
| *Bristowe, H. C., M.D. Lond. | Wrington, Somerset. |
| *Buckmaster, G. A., M.A., M.D. Oxon. | University of Bristol. |
| *Burdon-Cooper, J., M.D., B.Sc. Durh. | 12 The Circus, Bath. |
| *Burgess, R. | Somerset House, Canynge Rd., Clifton. |
| *Burnet, R. H., M.B., B.S. Durh. | 479 Bath Road, Brislington. |
| *Bush, J. Paul, *C.M.G.*, *C.B.E.*, Ch.M. Brist. | Vyvyan House, Clifton. |
| | |
| *Cairns, F. J. | Devon House, Whitehall Road, Bristol. |
| *Carey, R. S., *O.B.E.*, B.A. Cantab. | 502 Bath Road, Brislington, Bristol. |
| *Carleton, H. H., M.A., M.D. B.Ch. Oxon. | 1 Lansdown Road, Clifton. |
| *Carling, A., M.A., M.B., B.C. Cantab. | 12 Great George Street, Bristol. |
| *Carwardine, Thomas, M.B., M.S. Lond. | 16 Victoria Square, Clifton. |
| *Cave, Edward J., M.D. Lond. | 20 The Circus, Bath. |
| *Chambers, E. R. | 9 Clifton Park, Clifton. |
| *Charles, J. R., M.A., M.D., B.C. Cantab. | Deepholm, Clifton. |
| *Chitty, H., M.S. Lond. | 46 Pembroke Road, Clifton. |
| *Christofferson, P. E., M.B., Ch.B. Brist. | 8 Lansdown Place, Clifton. |
| *Claremont, L. E. | 5 Rodney Place, Clifton. |
| *Clarke, R. C., *O.B.E.*, M.B., Ch.B. Brist. | 29 Victoria Square, Clifton. |
| *Coates, Vincent M., *M.C.*, M.A., M.D B.C. Cantab. | 10 The Circus, Bath . |
| Connellan, P. S. | 12 Bloomsbury Street, London, W.C.1. |
| *Coombs, Carey, M.D. Lond. | 3 Pembroke Road, Clifton. |
| *Cooper, William Russell | 13 Westfield Park, Redland, Bristol. |
| *Corfield, C., M.D. Durh. | 5 Kensington Place, Clifton. |
| Cossham, W. R., M.D., C.M. Aberd. | Wellesley House, Cirencester. |
| Cridland, A. B. | Salisbury House, Chapel Ash, Wolverhampton. |
| *Cross, F. R., M.B. Lond., LL.D. Brist. | Worcester House, Clifton. |
| *Crossman, F. W., M.B. Durh. | White's Hill, Hambrook near Bristol. |
| *Crouch, C. P. | 5 Harley Place, Clifton. |
| | |
| *Dacre, John | 14 Eaton Crescent, Clifton. |
| *Datta, S., M.B., B.Ch. Bris. | Stapleton Institution, Bristol. |
| *Davies, E. D. D. | Standish House, Stonehouse, Glos |
| *Davies, D. S., M.D. Lond., D.P.H. Cantab. | 6 Lansdown Place, Clifton. |
| Devine, H., M.D Lond. | Corporation Mental Hospital, Portsmouth. |
| *Devis, H. F. | Shrubbery House, Shrubbery Road, Downend. |
| *Dix, C. | 11 Victoria Square, Clifton. |
| *Dixon, G. C., B.A., M.B. | Bristol Eye Hospital. |
| *Dixon, T. B. | 134 Coronation Road, Bristol. |
| *Drapes, T. L., M.B., B.C., B.A. Cantab. | St. Maur, Beaufort Square, Chepstow. |
| *Duerden, J. R.. M.B., Ch.B. Brist. | 149 Bell Hill Road, St. George. |
| *Dykes, A. L., M.D. Lond. | 7 Julian Road, Bristol. |
| Dymoke, F. | 21 Cotham Road, Bristol. |

| | |
|---|---|
| *Edgeworth, F. H., M.D., B.C., M.A.Cantab., D.Sc.Lond. | 13 Richmond Hill, Clifton. |
| *Eglinton, J. H. C. | Street, Somerset. |
| *Elkington, G. W., M.B., B.S. Lond. | Bruton, Somerset. |
| *Elliot Bros. Ltd. | *Australian Pharmaceutical Notes & News*, O'Connell Street, Sydney, N.S W , c/o Grimwade, Ridley & Co., 124 Minories, E.C. |
| Elliott, F. Percy, M.B., C.M. Aberd. | 113 Grove Rd., Walthamstow, London' E.17. |
| *Elliott, W. H. A., M.B., B.S. Lond. | 1 Upper Belgrave Road, Clifton. |
| *Every-Clayton, L. E. V., M.D. Lond | River Bank, Sunbury-on-Thames. |
| | |
| *Faill, C. J. C. | 19 Portland Square, Bristol. |
| Farnfield, W. W. | Clive Vale, Gillingham, Dorset. |
| *Fallon, Col. J. | 35 Cavendish Road, Henleaze, Bristol. |
| *Fawn, G. F. | 6 Oakfield Road, Clifton. |
| *Fells, A., M.B., C.M. Edin. | 6 Elton Road, Clifton. |
| *Firth, J. L., M.D., M.S. Lond. | 8 Victoria Square, Clifton. |
| *Flemming, A. L., M.B., Ch.B. Brist. | 48 Pembroke Road, Clifton. |
| *Flemming C. E. S. | Manvers House, Bradford-on-Avon. |
| *Foss, E. V., M.D., Ch.B. Brist. | Clouds Hill House, St. George, Bristol. |
| *Fraser, A. D., M.A., M.B., Ch.B. | 4 Alexandra Road, Clifton. |
| | |
| *Garden, R. R., M.A., M.B., B.Ch. | 9 Harley Place, Clifton. |
| Garland, E. B., M.B., C.M. Edin. | 114*a* Hampton Road, Redland, Bristol. |
| *Gee, C. A. H., M.B., Ch.B. Brist. | Portbury, Nr. Bristol. |
| *Gibbs, A. N. Godby | 30 Whiteladies Road, Clifton. |
| *Glenny, E. T., M.B., B.S. Lond. | 102 Pembroke Road, Clifton |
| *Golding, Madge E., M.B., Ch.B. | 76 Cranbrook Road, Redland. |
| *Gordon, R. G. M.D., B.Sc. Edin. | 9 Circus, Bath. |
| *Gornall, W. A., M.B., Ch.B. Brist. | 23 Windsor Road, St. Andrew's Park. |
| *Green, T. A., *D.S.O.*, M.D., C.M. Edin. | 33*a* Whiteladies Road, Clifton. |
| Griffiths, Cornelius A. | 35 Newport Road, Cardiff. |
| *Griffiths, J. S. | 20 Redland Park, Bristol. |
| *Griffiths, S. J. H., M.B., Ch.B. Brist. | Fairlawn, Portishead. |
| *Groves, E. W. Hey., M.D., M.S. Lond. | 25 Victoria Square, Clifton. |
| | |
| *Hadfield, G., M.D. Lond. | General Hospital, Bristol. |
| *Hailes Clements D. G., M.D., C.M. Ed. | 2 Richmond Park Road, Clifton. |
| *Hall, E. George, M.B. Lond. | 42 Apsley Road, Clifton, Bristol. |
| *Hall, I. Walker, M.D., Ch.B.Vict. | Manilla Lodge, Clifton. |
| *Ham, C. I., M.B., Ch.B. Brist. | Elm Lane, Bristol. |
| *Harris, H. Elwin, B.A., M.B. Cantab. | 13 Lansdown Place, Clifton. |
| *Harris, H. E., M.A., M.B., B.Ch. Cantab. | 13 Lansdown Place. |
| *Harsant, W. H. | Tower House, Clifton Down Road Clifton. |
| *Harty, J. P. I., M.B., B.Ch., B.A.O., R.U.I. | Deepholm, Clifton. |
| *Hawkins, E. J. | Constantine House, Avonmouth. |
| *Hector, F., M.D. Lond. | Ham Green Hospital, Pill. |
| *Herapath, C. E. K., *M.C.*, M.D. Lond. | Ormlie, Clifton. |
| *Hill, Hedley, M.D. Durh. | 101 Pembroke Road, Clifton. |
| Hospital, Bristol General (*Secretary*, T. W. Gregg). | |
| *Houghton, L. M., M.B., B.S. Lond. | 74 Church Road, Redfield. |
| Hughes, D. | 12 Cotham Road, Bristol. |
| *Hunter, F. S. | c/o Messrs. J. Wright & Sons Ltd., Publishers, Bristol. |

Iles, A. E., *O.B.E.*, M.B., B.S. Lond. ... ... 17 Victoria Square, Clifton.
Infirmary, Bristol Royal (*Secretary*, E. C. Smith).
Ingle, C. Durban ... ... ... ... ... ... ... Somerton, Somerset.

*Jackman, W. A. ... ... ... ... ... ... ... Mortimer Road, Clifton.
*Johnson, R. G., M.B., D.P.H. ... ... ... ... 119 Redland Road, Bristol.
*Joll, C. A., M.B., M.S. Lond. ... ... ... ... 64 Harley Street.

*Kennedy, W. P. M.D. Dubl. ... ... ... ... 3 Gay Street, Bath.
*Kemm, N. F., M.B., B.S. Lond. ... ... .. ... 12 Duchess Road, Clifton.
*Kerby, T. R. R. ... ... ... ... ... ... ... Heywood, Pill, Nr. Bristol.
*Kerfoot, Stanley J. ... ... ... ... ... ... 109 East Street, Bedminster, Bristol.
*Kingston, S. H., M.B., Ch.B. Brist. .. ... 3 Whiteladies Road, Clifton.
*Kyle, H. G., M.A., M.D. B.Ch. Oxon. ... ... 31 Westbury Road, Bristol.

*Lace, F. ... ... ... ... ... ... ... ... ... 5 Gay Street, Bath.
*Lavington, C C., M.B., B.S. Durh. .. ... 1 Burlington Road, Redland, Bristol.
*Lees, E. Leonard, M.D., C.M. Ed. ... ... ... 22 Richmond Hill, Clifton
*Legat, R. Eddowes M.B., C.M. Edin.... ... Murray House Staple Hill, Bristol.
Leigh, W. W. ... ... ... ... ... ... ... ... Llansamnor House, Cowbridge Glam.
*Leonard, R. C., M.D. Durham ... ... ... ... Bower Ashton, Bristol
*Levis, J. S., *M.C.*, M.B., B.Ch., N.U.I. ... 20 Gay Street, Bath.
*Linton, Marion S., M.B. Lond. ... ... ... 21 Oakfield Road, Clifton.
*Lister, A. E. J., Lt.-Col., I.M.S. (Retired) ... 12 All Saints Road, Clifton.
*Lister, L. Margaret ... ... ... ... ... 12 All Saints Road, Clifton.
*Logan, H. B. ... ... ... ... ... ... ... ... Eastfield, Southville, Bristol.
*Lucas, J. J. S., M.D. Lond. ... ... ... ... ... 186 Wells Road, Totterdown, Bristol.

*Macdonald, P. T. T., M.A., M.B., Ch.B. ... 43 Regent Street, Kingswood.
Mackinnon, Charles, M.B., C.M., M.A. Glasg. Davaar, Cirencester.
Maclean, Sir Ewen J., M.D., C.M. Ed. ... 12 Park Place, Cardiff.
*MacLeod, Donald, M.B., C.M. Ed. ... ... 84 Redland Road, Redland, Bristol.
*MacLeod, G., *M.C.*, M.D., Ch.B. ... ... ... "Wargrave," Clevedon.
*MacWatters, J. C., *M.B.E.* ... ... ... ... Almondsbury, near Bristol.
*McKenzie, W. G., *M.C.* ... ... ... ... ... 101 Coronation Road, Bristol.
*McLannahan, J. G. ... ... ... ... .. ... The Mount, Stonehouse.
Marshall, Arthur L., M.B., B.A. Cantab. ... Raveningham, Norwich.
*Mather, J. S., M.B., C.M. Aberd. ... ... ... Lawrence Hill House, Bristol.
*Mathew, W. E., M.D. ... ... ... ... ... ... 85 Coldharbour Road, Redland.
*Mayes, F. J. A. ... ... ... ... ... ... ... 79 Pembroke Road, Clifton.
Miall, C. L'O. ... ... ... ... ... ... ... Elm Hayes, Paulton, near Bristol.
*Milling, T., M.B., Ch.B. ... ... .. ... ... 1 Vicarage Road, Southville.
*Milner, A. N. P. ... ... ... ... ... ... ... 318 Stapleton Road, Bristol.
*Moore, C. A., M.B., M.S. Lond. ... ... ... 56 St. Paul's Road, Clifton.
*Morris, A. G. ... ... ... ... ... ... ... 18 Westbury Park, Westbury-on-Trym.
Morris, L. N., M.B., Ch.B. Brist. ... ... ... 24 All Hallows Road, Upper Easton, near Bristol.
*Morris, Mary E. H., M.D. Lond. ... ... ... 19 Gay Street, Bath.
*Morton, C. A. ... ... ... ... ... ... ... ... 14 Vyvyan Terrace, Clifton.
*Mumford, W. G., M.B. Lond. ... ... ... ... 18 The Circus, Bath.
*Murray, L. A. ... ... ... ... ... ... ... ... Fairfield, Waltor, Clevedon.
*Myles, G T. ... ... ... ... ... ... ... ... 7 Apsley Road, Clifton.

| | |
|---|---|
| *Neild, Newman, M.B., B.Ch. Vict. ... ... ... | 9 Richmond Hill, Clifton. |
| *Newnham, W. H. C., M.B., M.A. Cantab. ... | 3 Lansdown Place, Victoria Square, Clifton. |
| *Nichols, F. C., *M.C.*, M.B., Ch.B. Brist. ... | 38 Whiteladies Road, Clifton. |
| *Nixon, H. C., M.D. Edinb. ... ... ... ... | 5 Queen's Parade, Bath. |
| *Nixon, J. A., *C.M.G.*, M.D. Cantab. ... ... | 7 Lansdown Place, Clifton. |
| *Nott, Winifred, M.B., Ch.B., Brist. ... ... | "Fassiefern," Upper Cranbrook Road. |
| Oppenheimer, Son & Co. Ltd. ... ... ... | 179 Queen Victoria Street, London, E.C.4. |
| *Ormerod, H. Lawrence, M.D., B.Ch. R.U.I. | 2 Henleaze Road, Westbury-on-Trym, Bristol. |
| *Orr-Ewing, H. J. *M.C.* M.D. Lond. ... ... | English Mission Hospital, Jerusalem. |
| *Parker, George, M.D., M.A. Cantab. ... ... | 14 Pembroke Road, Clifton. |
| Parkes, J. A., M.B., Ch.B. Liverpool. ... ... | 2 Effingham Road, Bristol. |
| Parkinson, Major G. S., *D.S.O.*, R.A.M.C.... | R.A.M.C. Mess, Grosvenor Place, London, S.W. 1. |
| *Parsons, H. F. ... ... ... ... ... ... ... | The Heath, Hazelwood Road, Sneyd Park |
| Parsons, Sir J. H., M.B., B.S., D.Sc. Lond.... | 54 Queen Anne Street, London, W. |
| Paterson, Donald Rose, M.D., C.M. Ed. ... | 15 St. Andrew's Crescent, Cardiff. |
| *Phillips, J. R. P., *C.B.E.* ... ... ... ... ... | Northwoods, Winterbourne. |
| *Pollard, J., M.B., B.Ch., B.A.O. ... ... ... | 52 Cumberland Road, Bristol. |
| Powell, J. J., M.D. Lond. ... ... ... ... ... | 2 Gloucester Road, Bishopston, Bristol. |
| *Pridie, H. H., M.B., C.M. Ed. ... ... ... | 17 Oakfield Road, Clifton. |
| *Phillips, P., M.B., Ch.B. Brist. ... ... ... | Southmead Hospital. |
| *Rayner, D. C. ... ... ... ... ... ... ... | 9 Lansdown Place, Clifton, Bristol. |
| *Renton, R. A. S., *M.C.*, M.D. Durh.... ... ... | Tortworth House, Clevedon. |
| *Robertson, D., M.B. Ch.B. Edin. ... ... ... | 205 Wells Road, Knowle. |
| *Robertson, M. K., M.B., B.Ch. Cantab. ... | 17 Park Grove, Henbury. |
| *Rogers, Beatrice, ... ... ... ... .. ... | Richmond Hill, Clifton. |
| *Rogers, B. M. H., M.D., B.Ch., B.A. Oxon. ... | 14 Mortimer Road, Clifton. |
| *Rolfe, R., B.A., M.B., B.C. Cantab. ... ... | Park House, St. Andrews Road, Avonmouth. |
| *Rose, F. H. ... ... ... ... ... ... ... ... | 8 Chantry Road, Clifton. |
| *Rutherford, J. M., M.B., C.M. Edin. ... ... | Brislington House, near Bristol. |
| *Salisbury, Walter, M.D., M.S. Lond. ... | Oakroyd, Abbey Park Road, Grimsby. |
| Savage, W. G. ... ... ... ... ... ... ... ... | "Bourn," 7 Trewartha Park, Weston-super-Mare. |
| *Scott, H. H. ... ... ... ... ... ... ... ... | 78 Old Market Street, Bristol. |
| *Shaw J. E., M.B., C.M., Edinb. ... ... ... | 23 Caledonia Place, Clifton. |
| *Short, A. R., M.D., B.S., B.Sc. Lond. ... ... | 69 Pembroke Road, Clifton. |
| *Short, L. J., M.D., B.S. Lond., M.D. Brist. ... | Maywood, Atlantic Road South, Weston-super-Mare. |
| *Smith, G. Cockburn, M.D. Brux. ... ... ... | 12 South Road, Newton Abbot. |
| Smith, Rev. Reginald, M.A. Cantab. ... ... | The Vicarage, Walpole St. Andrew, Norfolk. |
| *Smith, W. Alexander M.B., M.A. Oxon. ... | 70 Pembroke Road, Clifton. |
| *Smyth, Richard, M.A., M.D. Dub. ... ... | Burnley, Westbury-on-Trym. |
| *Smythe, H. J. D., *M.C.*, M.B., M.S. ... ... | 72 St. Paul's, Road, Clifton. |
| *Spoor, A., M.B., B.Ch. Cantab.... ... ... ... | The Hydro, Bristol. |
| *Staniland, M. F. ... ... ... ... ... ... ... | 255 Stapleton Road. |

| | |
|---|---|
| *Statham, R. S. S., *O.B.E.*, M.D., M.Ch. Brist. | 24 College Road, Clifton. |
| *Stock, W. S. V., M.B., B.S. Lond., M.B., B.Ch. | 1 Mortimer Road, Clifton. |
| *Stewart, A. L., D.M., D.Ch. Brux. ... ... | 31 Howard Road, Southville. |
| *Stuart, Agnes, M.B., Ch.B. ... ... ... ... | Raleigh Road, Bedminster, Bristol. |
| *Symes, J. O., M.D. Lond ... ... ... ... ... | 71 Pembroke Road, Clifton. |
| | |
| *Tasker, D. G. C., M.B., M.S. Lond. ... ... | 16 All Saints Road, Clifton. |
| Taylor, A. L. ... .. ... .. ... ... ... ... | The Royal Infirmary, Bristol. |
| *Taylor, H. N., M.A. Cantab., M.D. Dub. ... | Hillside, Ebbw Vale. |
| *Temple, G. H., M.B., C.M. Ed. ... ... ... | Ailanthus, Weston-super-Mare. |
| *Templeton, W. L., M.B., Ch.B. Glasgow ... | 143 Cheltenham Road, Bristol. |
| *Terry, C. H., M.A., M.B., B.Ch. Oxon. .. ... | 15 Circus, Bath. |
| Thin, James ... ... ... ... ... ... ... ... ... | 54 & 55 South Bridge, Edinburgh. |
| *Thomas, J. D., M.B. Cantab. ... ... ... ... | Northwoods, Winterbourne. |
| *Todd, A. T., *O.B.E.*, M.B., Ch.B. Edin. ... | Clayton, Clifton Park, Clifton. |
| *Trist, J. R. R., *M.C.* ... ... ... ... ... ... | 120 Henleaze Road, Clifton. |
| *Tryon, V., M.B., Ch.B. Brist. ... ... ... ... | 3 Harley Place, Clifton. |
| | |
| *Vickery, W. H. ... ... ... ... ... ... ... ... | 1 Trewartha Park, Weston-super-Mare |
| | |
| *Walker, Cyril H., M.B., B.A. Cantab. ... ... | 8 Oakfield Road, Clifton. |
| Walker, H. B. ... ... ... ... ... ... ... ... | 12 The Avenue, Minehead. |
| *Wallace, John, *O.B.E.*, M.B., C.M. Edin. ... | 10 Knightstone Road, Weston-super-Mare. |
| *Walters, C. F. ... ... ... ... .. ... ... | 5 Mortimer Road, Clifton. |
| *Wanklyn-James, C. W., *O.B.E.* ... ... ... | 3 Mortimer Road, Clifton. |
| *Warren, R., M.A., M.D. Oxon. ... ... ... | 1 Royal Terrace, Weston-super-Mare. |
| *Waterhouse, R., M.D. Lond. .. ... ... ... | 25 Circus, Bath. |
| *Watson-Williams, P., M.D. Lond.... ... ... | 2 Rodney Place, Clifton. |
| *Watson-Williams, E., *M.C.*, M.B., B.Ch., B.A. Cantab. ... ... ... ... ... ... ... | 12 Victoria Square, Clifton. |
| *Weston, B. A. A., M.B., B.Ch. Brist. ... ... | 30 Cotham Grove, Bristol. |
| *Whicher, A. H. ... ... ... ... ... ... ... ... | 115 Queen's Road, Clifton. |
| Whitehouse, H. Beckwith, M.S. ... ... | 52 Newhall Street, Birmingham. |
| Wigan, C. A., M.D. Durh., J.P.... ... ... ... | Deepdene, Portishead. |
| Wigmore, J., M.D. R. U. I.... ... ... ... ... | 16 Queen Square, Bath. |
| *Willcox, J. W. J., M.B., B.S. Lond. ... ... | Glastonbury. |
| Willcox, R. Lewis ... ... ... ... ... ... ... | 97 London Road, Salisbury. |
| *Williams, E. C., M.B., B.C., B.A. Cantab. ... | 159 Whiteladies Road, Clifton. |
| *Williams, G. C., B.A. Cantab. ... ... ... | Town Hall, Oxford. |
| *Williams, L. H., M.D. Durh.... ... ... ... | Thornbury, Glos. |
| Williams, S. R., M.B. Lond. ... ... ... ... | Buckfastleigh, Devon. |
| *Wills, W. K., M.B., B.C. Cantab. ... ... ... | 19 Whiteladies Road, Clifton. |
| *Wood, Duncan ... ... ... ... ... ... ... ... | 5 Eaton Crescent, Clifton. |
| *Wood, W. V., *M.C.* ... ... ... ... ... ... | Henley Lodge, Yatton, Som. |
| *Wright, A. J., M.B., B.S. Lond. ... ... ... | 14 Victoria Square, Clifton. |

